# Bioprinting High‐Cell‐Density Cardiac Tissue Constructs With Sustained Contractile Function

**DOI:** 10.1002/adhm.71446

**Published:** 2026-07-19

**Authors:** Priscila Melo, Wing Tai Tung, Harvey Lewis, Kavin Hettiarachchilage, Babis Tzivelekis, Rolando Berlinguer‐Palmini, Ana Marina Ferreira, Jason Gill, Kenneth Dalgarno

**Affiliations:** ^1^ School of Engineering Newcastle University Newcastle Upon Tyne UK; ^2^ Bio‐Imaging Unit Medical School Newcastle University Newcastle Upon Tyne UK; ^3^ School of Pharmacy and Translational and Clinical Research Institute Faculty of Medical Sciences Newcastle University Newcastle Upon Tyne UK

**Keywords:** cardiotoxicity, contractility, dysrhythmia, electrophysiology, reactive jet impingement

## Abstract

The development of in vitro models that reflect key elements of cardiac physiology remains a major challenge for assessing cardiotoxicity and drug efficacy. Tissue engineering approaches, together with advanced biofabrication technologies, provide a new way to recreate the target tissue composition and organization, enabling the development of 3D in vitro models able to mimic the biological function of the target tissue. Here, we present a novel 3D bioprinted cardiac in vitro model fabricated using reactive jet impingement (ReJI), an advanced biofabrication technique that enables rapid, high‐resolution deposition of living cells within a bespoke hydrogel matrix composed of type I collagen, alginate, and fibrin. Using the murine HL‐1 cardiomyocyte cell line, we demonstrate for the first time that ReJI bioprinting supports the formation of stable, functional 3D cardiac constructs exhibiting spontaneous and sustained contractility over 21 days, without external stimulation. The 3D models expressed key biomarkers related to cell contraction: desmin, myosin, and gap junction alpha‐1 (GJA1). In particular, cultures showed increased early‐stage expression of GJA1, indicative of the formation of gap junction structures within the 3D culture, and were susceptible to drug‐induced dysrhythmia, confirming their significant potential for use in cardiotoxicity testing.

## Graphical abstract

Contractile cardiomyocytes in 3D culture provide insights into the behavior of the heart. Combining a 3D bioprinting approach with a multi‐electrode array allows the development of the 3D culture to be monitored over time, and matured cultures allow the effect of drugs on cardiac function to be modelled, replicating effects seen in clinical application.

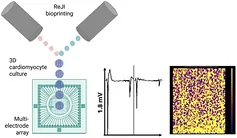

## 1 Introduction

Cardiotoxicity remains one of the principal reasons for late‐stage drug attrition and post‐market withdrawal, underscoring the necessity of preclinical models that can reliably identify detrimental cardiac responses earlier in the drug development cycle [1, 2, 3, 4]. Two‐dimensional (2D) in vitro models are considered standard practice, and within cardiac toxicology studies, such models have demonstrated their value in identifying drug‐induced proarrhythmic liabilities, applied across a number of screening platforms. These platforms enable the measurement of action potentials, intracellular calcium flux, and contractility in a controlled environment, allowing direct observation of drug‐cardiomyocyte interactions. Despite their utility, 2D monolayer cultures lack the structural and mechanical complexity of native myocardium. The absence of three‐dimensional (3D) cell–cell and cell–matrix interactions limits cell maturation and electrophysiological anisotropy, resulting in an immature phenotype and simplified tissue‐level responses [5]. In 3D configurations, cardiomyocytes display enhanced sarcomere organization, improved calcium handling, and more physiologically relevant contractile force generation. Moreover, these constructs facilitate the inclusion of non‐myocyte populations such as fibroblasts and endothelial cells, promoting the paracrine and biomechanical cues necessary for tissue homeostasis and realistic screening results with clear translational impact. Importantly, 3D cultures offer an opportunity for multi‐parametric endpoints, ranging from electrophysiological mapping to extracellular matrix remodeling, that are not feasible in simpler monolayer systems.

Among emerging 3D approaches, bioprinted cardiac constructs represent a particularly promising advancement. Unlike manually assembled engineered tissues, bioprinting allows high reproducibility and scalability, attributes essential for standardizing cardiotoxicity assessments within screening pipelines [6]. Additionally, these models align with the ongoing regulatory need for more clinically representative assays, as reflected in updates to ICH S7B/E14 and the FDA's Predictive Toxicology Roadmap [7].

**FIGURE 1:**
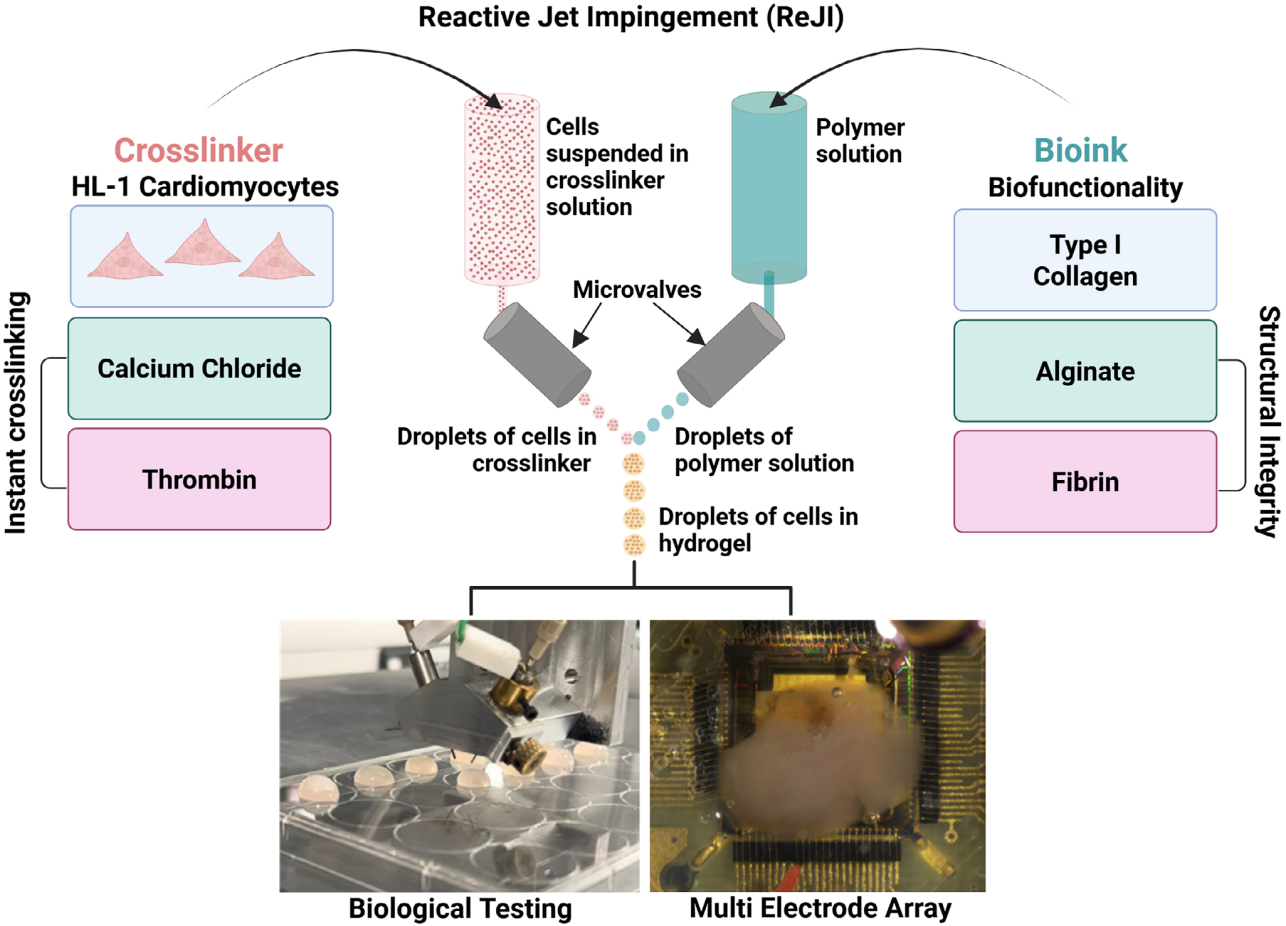
ReJI bioprinting of HL‐1 cells into 3D cardiac tissue constructs using CAF as a bioink. Two types of samples were bioprinted: Gel on a TCP substrate for standard biological testing and Gel on HD‐MEA‐Accura‐3D chips for electrophysiology measurement. Prepared in part using Biorender.

Bioprinting systems are generally classified as jetting, extrusion, or vat polymerization [8]. Jetting approaches have historically focused on the use of inkjet and microvalve technologies to print cells in media or low‐viscosity solutions. Extrusion and VAT polymerization approaches tend to focus more on the creation of 3D hydrogel cultures, which offer hydrated 3D structures which can chemically and mechanically mimic extracellular matrix [9]. Extrusion‐based bioprinting can impose a compromise between printability and cell viability as high viscosity bioinks cause damaging shear stresses within the hydrogel [10], and volumetric vat polymerization processes bring a number of optical constraints which limit choice of resin and scalability [11]. Within this study, we use reactive jet impingement (ReJI) bioprinting [12], which is a jetting‐based approach which can produce 3D hydrogel cultures. The process is illustrated in Figure 1: two solutions are used, one a bioink solution and the other a crosslinking solution with cells in suspension. Two microvalves (arranged in a V configuration) generate droplet streams of each both solutions, with the droplet streams defined such that the droplets collide in mid‐air. When the droplets collide the fluids react to form the hydrogel, embedding the cells within the hydrogel droplet which then falls to the substrate. Because the microvalves process low viscosity hydrogel precursor solutions shear stresses in the bioinks are low [13], and so the printability/cell viability trade‐off for ReJI is less limiting than for extrusion bioprinting, but with the constraint that a reactive material system is required. The ReJI process allows for printing onto a wide range of substrates, including hard, delicate, and rough substrates [14, 15], and here we exploit this by directly printing 3D cardiomyocyte cultures onto multi‐electrode array (MEA) chips. This combines the ability to create a functional cardiac tissue construct with sustained contractility together with electrophysiological characterization which can detect changes in rhythm in response to the introduction of specific compounds. We demonstrate that this gives a tractable and scalable approach to electrophysiological characterization of cardiac constructs.

## 2 Materials and Methods

### 2.1 Materials

All cell culture materials were obtained from Sigma‐Aldrich, unless stated otherwise, including sodium alginate (180947), fibrinogen from bovine plasma (F8630), thrombin (T4648, 10K units), calcium chloride (CaCl_2_, C1016), Dulbecco's Phosphate Buffered Solution (DPBS, D8537), EmbryoMax gelatin solution (ES‐006, 0.1%), fibronectin from bovine plasma (F1141, 1 mg/mL), L‐ascorbic acid (A4544), ultrapure water (TMS‐011), (±)‐norepinephrine (+)‐bitartrate salt (A0937), Claycomb basal medium (51800C), HL‐1 cell screened fetal bovine serum (TMS‐016), goat serum (S26‐M), Tween 20 (P1379), Triton‐X100 (X100), monoclonal anti‐desmin antibody produced in mouse (D1033), 4,6‐diadmidino‐2‐phenylindole (DAPI, MBD0015), and dimethyl sulfoxide (DMSO, D2650).

### 2.2 Formulation of the Collagen‐Alginate Acid‐Fibrinogen Bioink

The formulation of the CAF bioink has been previously reported [7, 8]. sodium alginate was added to calcium‐ and magnesium‐free DPBS to a concentration of 2.5 wt%. and stirred at 35°C to achieve full dissolution. Fibrinogen was dissolved in DPBS and slowly stirred at 35°C to an initial concentration of 10 wt%, which was then further diluted in DPBS to a concentration of 3.7 wt%. For crosslinking, a 6 wt%. CaCl_2_ solution was prepared in DPBS at room temperature. Thrombin was diluted in Dulbecco Modified Eagle Medium (DMEM; 11966025, Gibco, UK) to a concentration of 500 U/mL. The final bioink was prepared by mixing commercial type I bovine collagen solution (6 mg/mL, FS22052, Collagen Solutions, UK), sodium alginate and fibrinogen solutions, in a ratio of 1:2:8, respectively. The bioink was then filtered using a 100 µm cell strainer to remove large agglomerates. For the crosslinker solution, thrombin 500 U/mL was mixed with 6 wt%. CaCl_2_, leading to a final concentration of 0.1 vol% CaCl_2_.

### 2.3 Culture of HL‐1 Cardiomyocytes and Suspension in Bioink

The murine cardiac HL‐1 cell line (SCC065, Millipore, CVCL_0303) was used for a maximum of 15 passages to avoid phenotypic drift. Culture flasks were coated with a gelatin/fibronectin solution, comprising 0.02 vol% gelatin and 0.0005 wt% fibronectin, and incubated at 4°C overnight. Cells were cultured in Claycomb basal media supplemented with 10 vol% fetal bovine serum, 2 mM L‐glutamine (25030‐024, Gibco), 0.1 mM norepinephrine (in 30 mM ascorbic acid), and 1 vol% penicillin/streptomycin (15140‐122, Gibco) [9]. Cells were maintained in a humidified 5% CO_2_ atmosphere at 37°C. Before printing, cells were detached using 0.1% trypsin/EDTA (25200‐056, 0.25%, Gibco, diluted with DPBS), centrifuged at 300x*g* and resuspended in thrombin/CaCl_2_ crosslinking solution. For printing, the media‐to‐cell ratio in the crosslinking solution was adjusted to achieve a concentration of 5 x 10^6^ cells/mL of CAF hydrogel (Gel).

### 2.4 Bioprinting of HL‐1 Cardiomyocytes in CAF Hydrogels

Samples were printed using a ReJI bioprinting system, equipped with a pair of INKX0514950A micro solenoid valves (The Lee Company, USA). The CAF bioink precursor solution and the crosslinking solution with HL‐1 cells in suspension were held in two separate pressurized cartridges, each connected to one of the microvalves. For assessment of cytocompatibility, cell morphology, and gene expression, HL‐1‐laden hydrogels were bioprinted using 200–400 Hz jetting frequency, 1000 µs pulse width, and a pressure range of 450–480 mmHg for CAF bioink and 250–300 mmHg for the HL‐1/thrombin/CaCl_2_ crosslinking solution.

Samples were created by depositing droplets of polymerized material, each with a volume of 0.3 µL, and a cell density of 5 × 10^6^ cells/mL of CAF (Gel), onto uncoated tissue culture plastic (TCP). After printing, the 150 µL hydrogels were incubated in complete Claycomb media at 37°C in a 5% CO_2_ humidified atmosphere. As control, HL‐1 cells were manually seeded into gelatin/fibronectin‐coated 12‐well plates, at a seeding density of 125,000 cells/well. All samples were seeded in triplicate for each condition and analyzed at day 1, 3, and 7 (short‐term culture), to benchmark the results against existing work on HL‐1‐laden hydrogels [18, 19, 20, 21]. The culture was then extended to 21 days (long‐term culture), to evaluate the stability of the bioprinted constructs as well as their contractility and biological function across extended culture periods.

For electrophysiological characterization, 3D bioprinted constructs were fabricated by sequentially depositing layers of CAF hydrogel onto high‐density MEA chips (HD‐MEA Accura‐3D, 3Brain AG, Switzerland), covering an area of approximately 4 × 4 mm. The optimized ReJI printing parameters described above were used, with a maximum time between printing of subsequent layers of 3 min. Five layers were printed across electrode area, with a total hydrogel volume of 150 µL. The MEA chips consisted of 4096 individual electrodes, each 21 × 21 µm in size and 90 µm in height, with a 60 µm center‐to‐center spacing, enabling electrode penetration into the 3D gel and the recording of local field potentials throughout the construct's volume.

For calcium imaging assays, constructs were printed directly into Petri dishes using the same processing conditions. The 2D control cultures were generated by seeding HL‐1 cells onto gelatin/fibronectin‐coated Petri dishes or 2D MEA chips (Accura, 3Brain AG), which featured a similar electrode arrangement but with flat, surface‐level contacts. Cells were seeded at a notional density of 330 cells/mm^2^ of area in the chip/dish, equivalent to the TCP culture density, ensuring comparability across experimental conditions.

To assess drug‐induced modulation of cardiac electrophysiology and dysrhythmia, HL‐1 cells were bioprinted as above but with a x–y pixel spacing of 750 µm, and printing pressures at 380 mmHg for Gel, and 300 mmHg for both the cell suspension and crosslinker solutions. Constructs were printed directly onto HD‐MEA chips, in this case covering half the chip area, with three layers printed and a total hydrogel volume of 45 µL, with the smaller volume used to ensure rapid diffusion of compounds to the HL‐1 cells.

### 2.5 Biological Characterization of 3D Constructs: Viability, Morphology, and Genetic Expression

#### 2.5.1 Cell Viability and Survival

Cell viability was assessed with the Presto Blue assay (A13261, Invitrogen, Thermo Fisher Scientific), at days 1, 3, and 7, prepared according to manufacturer instructions. Briefly, cell culture media was aspirated and replaced with 10% PrestoBlue cell viability reagent diluted in complete Claycomb media, incubated for 2 h at 37°C in a humidified atmosphere of 5% CO_2_ (protected from light). The supernatant was transferred into a 96‐well plate and the fluorescence measured at an excitation of 560 nm and emission of 590 nm (FLUOstar Omega Microplate Reader; BMG Labtech). Cells were washed twice with DPBS, fresh medium was added, and the cells returned to 37°C in a humidified atmosphere of 5% CO_2_. Cell viability values were determined and documented using GraphPad Prism 9 software (GraphPad Software, La Jolla, CA, USA), representing data from 3 technical replicates.

Cell survival was evaluated using live/dead viability kit (L3224, Invitrogen), at days 1 and 3. The staining solution was prepared by mixing 2 µL of calcein with 4 µL ethidium bromide in 1 mL of DPBS. Media was removed from each well and the samples washed with DPBS, followed by incubation in live/dead solution for 30 min at 37°C in a humidified 5% CO_2_ atmosphere (protected from light). The solution was removed, the samples washed with DPBS, and then imaged using confocal microscopy (Zeiss LSM 800, GmBH). The collected micrographs were treated using the Zen software (Zeiss, GmBH).

#### 2.5.2 Immunofluorescence and Scanning Electron Microscopy (SEM)

Immunofluorescence staining was used to confirm the presence of specific cardiac biomarkers, sarcomeric desmin and myosin, after 1, 3, and 7 days of culture. At each time point, medium was removed, cells were washed twice with DPBS, followed by fixation with 4 vol% paraformaldehyde (PFA, K19943‐K2, Thermo‐Fisher) solution for 30 min, at room temperature. Cell membrane permeabilization was achieved by incubation in 0.1 vol% Triton‐X100 in DPBS for 3 min, followed by washing in DPBS. Non‐specific antibody binding was inhibited using 10 vol% normal goat serum (in 0.1 vol% Tween 20 in PBS) for 20 min. Samples were incubated for 1 h with primary antibodies diluted in DPBS containing 0.1% Tween 20 and 10% goat serum; anti‐sarcomeric myosin (MF20, 14‐6503‐95, Thermo‐Fisher, AB_2865202) diluted to 1:1000, or anti‐desmin diluted 1:40 (D1033, Sigma‐Aldrich, AB_476897). After washing in DPBS, cells were incubated for 1 h with Alexa Fluor 488 conjugated goat anti‐mouse IgG (A11001, Thermo‐Fisher, AB_2534069) diluted to 0.067 vol% in DPBS/Tween 20. After washing in DPBS, samples were immersed in a 1:1000 dilution of Alexa fluoro‐555 conjugated phalloidin/DPBS solution (A34055, Thermo‐Fisher) for 30 min at room temperature to stain the cytoskeleton. Finally, samples were incubated in 1:2500 DAPI/DPBS solution for 1 h to label cell nuclei. All steps were performed at room temperature. Samples were imaged with confocal microscopy as described in 2.5.1.

The samples for SEM analysis were collected on days 1 and 3. First, the medium was removed, and samples were washed with DPBS. Fixation was performed by immersing the gels in a solution of 2 vol% glutaraldehyde (TAAB Laboratory and Microscopy) for 10 min, followed by two 15 min washes in Sorensen's Phosphate Buffer (TAAB Laboratory and Microscopy). Samples were then dehydrated via sequential immersion in EtOH for 30 min at different concentrations, 25%, 50%, and 75%. To finalize, samples were immersed for 1 h in 100% EtOH. This step was repeated twice before storage in EtOH at 4°C. Prior to SEM observation, the gels underwent critical point drying with CO_2_ in a Baltec Critical Point Dryer (Leica Geosystems Ltd). Samples were subsequently mounted on aluminum stubs, fixed in place with Acheson's Silver Dag (Agar Scientific), and then dried overnight. Imaging was collected on a Tescan Vega LMU SEM (Tescan, Czech Republic), housed within EM Research Services, Newcastle University. The images were collected with the equipment's software, ATLAS.

#### 2.5.3 Real‐Time Quantitative Polymerase Chain Reaction (RT‐qPCR)

At each time point, samples were washed in RNA‐free water and frozen at −20°C for 24 h, then kept at −80°C until analysis. Samples were digested using Dispase solution (07923, Stem Cell Technologies), at 37°C overnight. The RNA was extracted using RNeasy Mini Kit (74106, Qiagen, Hilden, GmBH) following the manufacturer's protocols. Extracted RNA concentration was measured using a Nanodrop (ND‐100, Nanodrop Technologies, USA) and then added to the reverse transcription master mix (4368813, Thermo‐Fisher) to synthesize complementary DNA (cDNA), achieved using a thermocycler (2,720 Thermal Cycler, Applied Biosystems, USA) based on cycles of 10 min at 25°C, 120 min at 37°C, and 5 min at 85°C. RT‐qPCR was performed using TaqMan Fast Advanced Master Mix (4444557) and TaqMan probes (4331182, Thermo Fisher Scientific, UK) with the following genes, NPPA(Mm0125577_g1), NPPB(Mm01255770_g1), TnnT2(Mm01290256_m1), and GJA1(Mm01179639_s1). Gene expression raw data were acquired using QuantStudio 7 Real‐Time PCR System (Thermo Fisher Scientific, USA). The expression of the genes of interest was normalized to Glyceraldehyde‐3‐phosphate dehydrogenase (GAPDH, Mm99999915_g1) and presented as a relative expression using the 2^−(ΔΔCt)^ method, where TCP samples were used as a reference to calculate the fold ratio on short‐term cultures. For long‐term cultures, gene expression was evaluated using day 1 Gel samples as a reference.

### 2.6 Calcium Fluctuation Analysis

All samples were maintained at 37°C in a 5% CO_2_ humidified atmosphere and analyzed on days 1, 3, and 7. Before analysis, culture medium was removed, and samples were gently rinsed with Tyrode's buffer. Cultures were analyzed using the Fluo‐4 NW AM Calcium Assay Kit (F36206, Invitrogen, UK), prepared according to the manufacturer's instructions, incubated for 30 min at 37°C, in a 5% CO_2_ humidified atmosphere. After incubation, samples were rinsed, and fresh HEPES buffer was added. Calcium flux was recorded using a Nikon FN1 Eclipse fixed‐stage fluorescence microscope (Nikon, Tokyo, Japan) equipped with a BSiExpress camera. The acquired data was processed using a Chroma 49003 ET‐EYFP bandpass filter cube and analyzed using the NIS‐Element software. For each sample, 3‐min time‐lapse recordings were obtained. The resulting videos were analyzed using Nikon Elements‐JOBS software to quantify mean fluorescence intensity. Ten regions of interest (pixels showing calcium flux changes) were selected per field, and their relative fluorescence units (RFU) were corrected by subtracting local background intensity as per the equation below:

$$\mathrm{Relative}\;\mathrm{Fluorescence}\;\left(\mathrm{Frel}\right)=F_{\mathrm{mean}}-F_{b}$$

where *F*_mean_ indicates the mean fluorescence detected on the ten selected regions, and *F_b_* is the baseline intensity recorded. The averaged values were used to compare calcium activity between 2D and 3D cultures.

For the constructs cultured over 21 days (long‐term culture), quantitative analysis of calcium fluctuation from HL‐1 cells’ electrical activity was assessed using the Fluo‐4 Direct Calcium Assay Kit (F10471, Invitrogen). Before loading the dye, each sample was supplemented with 100 µL of fresh culture medium and incubated for 1 h at 37°C in a 5% CO_2_ atmosphere. Subsequently, 100 µL of the working dye solution was added to each sample, with incubation under the same conditions for 1 h, protected from light. The dye‐containing medium was then replaced with HEPES‐buffered Tyrode's solution to maintain physiological ionic conditions during imaging. Live‐cell calcium fluctuations were recorded using an EVOS M5000 fluorescence microscope (Thermo Fisher Scientific, USA). Real‐time imaging was performed at 30 frames per second (fps), with video output projected to a monitor and recorded via a video capture card using OBS Studio software. Recorded videos were converted to TIFF image sequences and processed using ImageJ. Regions of interest (ROIs) larger than 200 µm^2^, which showed changes in fluorescence intensity were selected, with three ROIs selected per gel, and three gels per time point. Within each region of interest (ROI), mean and minimum fluorescence intensities were extracted across all frames. Fluorescence fluctuations (ΔF/F_0_) were calculated as:

$$\Delta F/F_{0}=\left(F-F_{0}\right)/F_{0}$$

where *F* is the instantaneous fluorescence intensity, and *F_0_* is the regional lowest baseline intensity from the recorded duration. The resulting data were analyzed and visualized using GraphPad Prism (v9.0) to determine beating frequency and calcium flux amplitude over time.

### 2.7 In Vitro Recording of Electrical Activity

Electrophysiological activity of HL‐1 cardiomyocytes was recorded using the BioCAM DupleX MEA system (3D Brain AG, Switzerland) to monitor spontaneous electrical activity. Figure S1 gives an overview of the process. The sampling rate was 19753.78 Hz, with a hardware high‐pass filter cut‐off at 20 Hz and hardware high‐pass filter order of 1. Signals were acquired from all electrodes simultaneously and processed using BrainWave5 software (3D Brain AG, Switzerland). All data received on the electrodes were recorded during the experiment and processed after the experiment. The cardiac field potential was detected using the detection function provided in BrainWave 5, in which the filter setting is in Cutoff filter mode, bandpass filter with low Cut‐off frequency at 1 Hz and high cut‐off frequency at 100 Hz, using class FIR with the order 10, FFT method, and Rect Window, and the parameters for identifying the beat used hard threshold at −150uV, pre‐ and post‐peak wave duration at 50 and 450 ms, Q detection start (Pre‐peak) at 20 ms, T detection start (Post‐peak) at 75 ms, and refractory period of 40 ms. Values were averaged per active electrode. Each measurement session began with 5 min of stabilization in HEPES‐buffered medium, followed by 5 min of continuous recording.

For the evaluation of drug‐induced dysrhythmia, constructs were maintained in culture for 7 days, with daily medium exchange. On day 7, the MEA chips containing the 3D constructs were immersed in HEPES‐buffered Tyrode's solution (J67607.K2, Thermo‐Fisher) to establish baseline field potentials. Subsequently, constructs were sequentially exposed to 100 nM isoproterenol (I6504, Sigma‐Aldrich), a β‐adrenergic receptor agonist, for 30 min, followed by three 5 min washes and two 15 min washes, both with Tyrode's buffer. Treatment with 50 nM E‐4031 (M5060, Sigma‐Aldrich), a selective hERG potassium channel blocker, was performed by incubating constructs for 30 min in the compound solution. All recordings were captured for 60 s and processed using BrainWave 5 software (3Brain AG) to quantify field potential rate (fp/min) and evaluate drug effects on electrical activity.

### 2.8 Statistical Analysis

All quantitative tests were performed in triplicate for each sample, and results were presented as mean value ± standard deviation or standard error. Two‐way ANOVA with Bonferroni post‐hoc test was performed with GraphPad Prism 9 software (GraphPad Software, USA) using a level of significance of *p* < 0.05 (*), *p* < 0.01 (**), *p* < 0.001 (***), and *p* < 0.0001 (****).

## 3 Results

### 3.1 Characterization of Short‐Term Cultures: Benchmarking Cell Behavior of ReJI Bioprinted HL‐1 Laden Constructs

#### 3.1.1 Maintenance of Cell Viability and Survival

**FIGURE 2:**
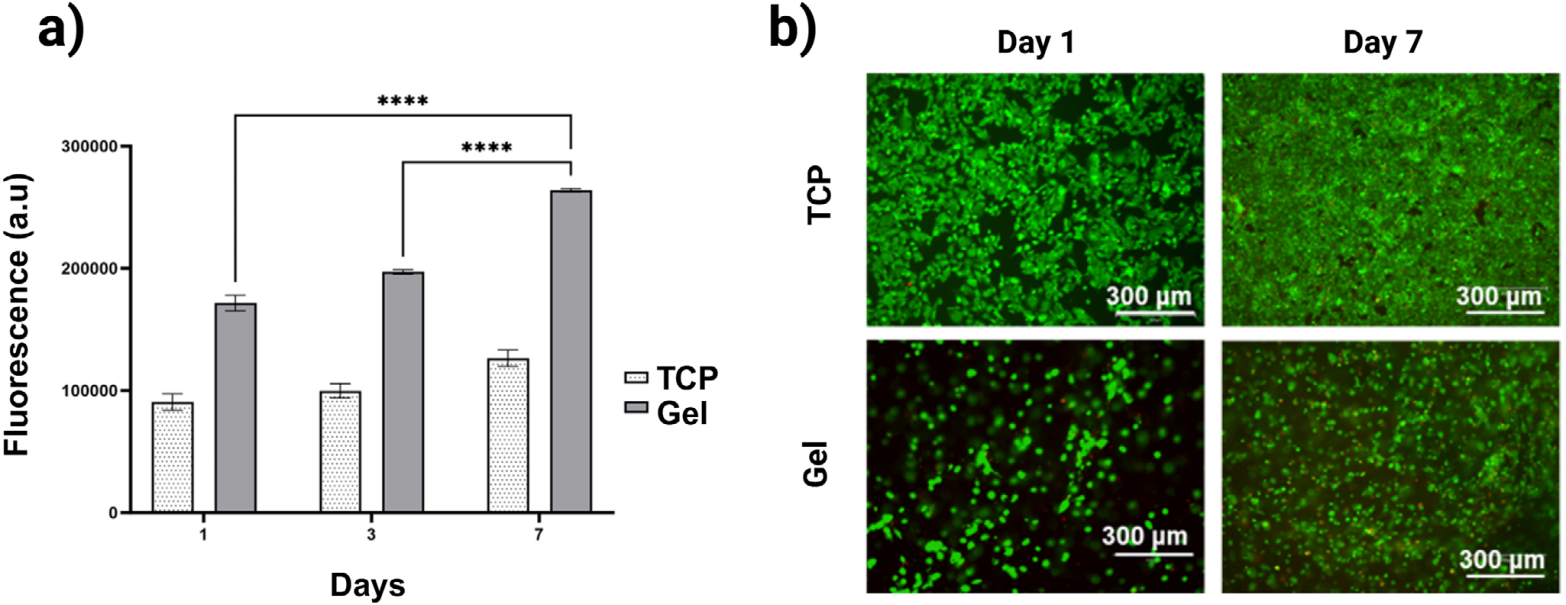
Assessment of HL‐1 cells' viability when seeded onto tissue culture plastic (TCP) and bioprinted in CAF hydrogel using ReJI (Gel), during 7 days of culture. (a) Statistically significant increases in cell viability are observed for printed hydrogels; *n* = 3; (****) *p*<0.0001. (b) Live/dead images depicting the presence of live cells (green, GFP channel) for all samples, across both time points, with little to no dead cells present (red, RFP channel). Separated red/green channels shown in Figure S2.

Cells embedded in the Gel showed a sustained increase in cell metabolic activity across all time points (Figure 2a), while it remained stable for cells culture on TCP. Cell survival assays (Figure 2b) revealed that live cells were dispersed well throughout the constructs and on TCP, remaining homogeneously distributed in both conditions up to day 3 of culture. HL‐1 cells typically show a cuboidal rather than elongated morphology in both 2D and 3D culture [22]. Together, this confirms that CAF is non‐cytotoxic for HL‐1 cells and the ReJI process did not induce stress severe enough to impair mitochondrial or enzymatic activity measured by the assay. Additionally, it supports the greater capacity for cellular growth, survival, and viability of HL‐1 cells in 3D, as opposed to 2D culture.

#### 3.1.2 Confirmation of Expression of Cardiac Markers

**FIGURE 3:**
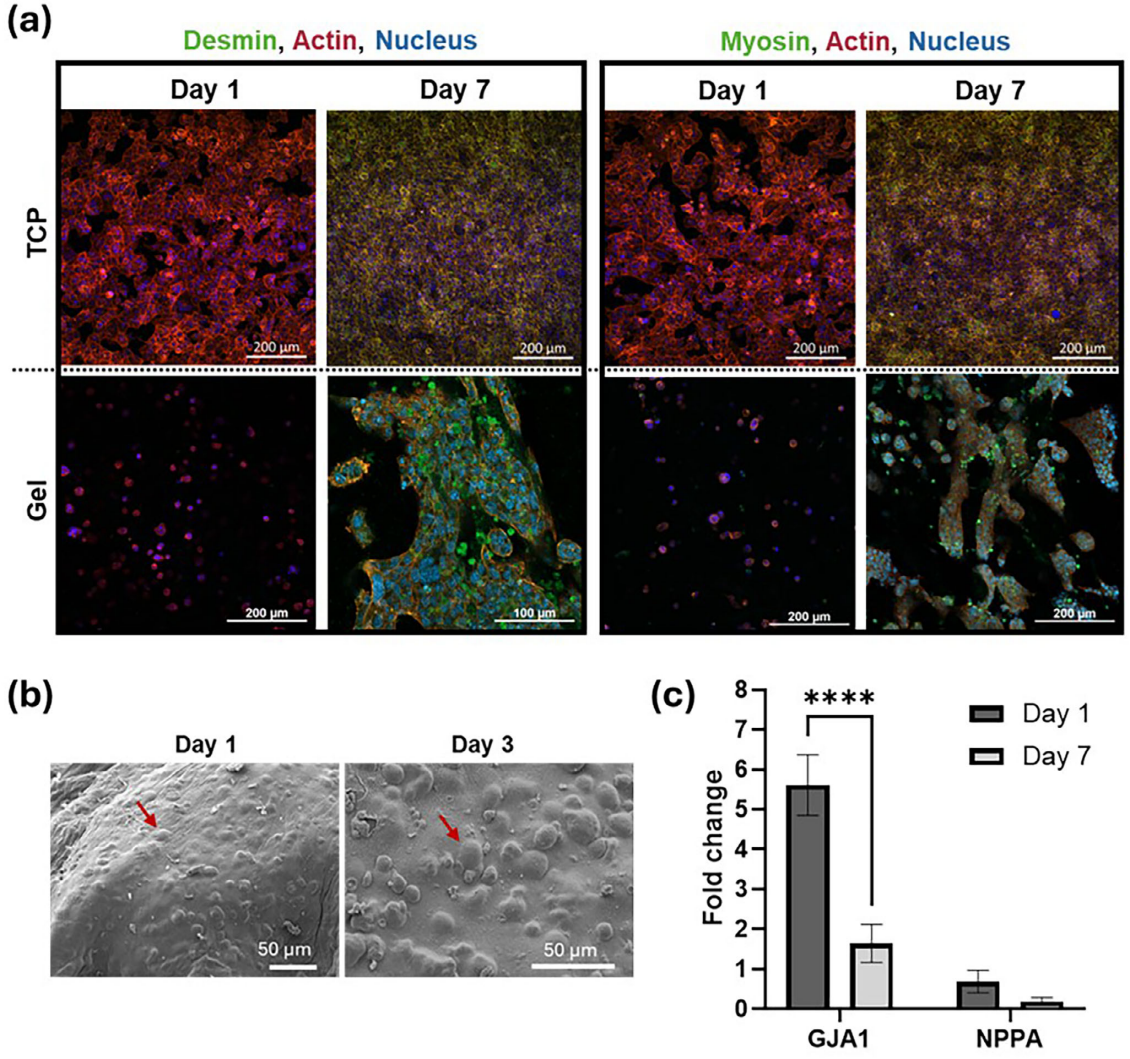
HL‐1 contractile cardiomyocytes cultured within Gel samples and in TCP. (a) Immunohistochemistry of HL‐1 contractile cardiomyocytes cultured within the gels and in the control (TCP). The predominant cardiac markers desmin and heavy chain myosin expressions were confirmed (green) as well as the expected atrial cell morphology (red, phalloidin) and large nucleus (blue, DAPI). Separated channels shown in Figure S3. (b) Morphology of HL‐1 cells cultured in the gel after 1 and 3 days, obtained via SEM, depicting the typical expected cell diameter of ∼ 20 µm [13]. (c) Confirmation of the expression of relevant cardiac markers GJA1 and NPPA in Gel samples, using TCP as a reference. Upregulation of GJA1 was seen for all time points, while NPPA remained low; *n* = 3; (****) *p* <0.0001.

The morphology of the cardiomyocytes was studied via confocal microscopy (Figure 3a) and SEM (Figure 3b). Staining of F‐actin in the cytoskeleton confirmed the expected HL‐1 morphology with a single centrally positioned nucleus, for all time points [23]. Despite the initial isolation of cells in the Gel, by day 7, spreading and interconnection between cells are observed, confirming cell recovery and construction of a putative syncytium. The expression of specific cardiac biomarkers was evaluated using immunofluorescence and RT‐qPCR (Figure 3a,c). Cardiac‐specific myosin and muscle‐specific desmin intermediate filaments were present, for both TCP and Gel samples, at day 7, with greater prevalence in the Gel samples (Figure 3a).

Maintenance of a cardiac phenotype was evaluated by analysis of natriuretic peptide precursor‐A (NPPA) and Connexin 43/Gap junction 1 (Cx43/GJA1) gene expression, using the 2D culture (TCP) as a reference [17]. Cardiomyocyte expression of Cx43/GJA1 is associated with the formation of gap junction structures, so early increased expression is a positive sign for the maturation of 3D cardiac cultures. Cx43/GJA1 remained upregulated from day 1, with a 6‐fold change reported compared to TCP, lowering to ∼ 2‐fold by day 7. NPPA is a gene expressed in atrial cardiomyocytes, such as the HL‐1 cells, and is involved in mediating homeostasis. A downregulation of NPPA was observed across the 7 days of culture, as previously observed [17]. Expression was at a similar level for both TCP and Gel at day 1, with a slight downregulation at day 7 in Gel samples; however, not statistically significant. This is an encouraging outcome since the upregulation of NPPA is associated with cardiac stress [24].

#### 3.1.3 Confirmation of Cellular Contractility via Intracellular Calcium Mobilization

**FIGURE 4:**
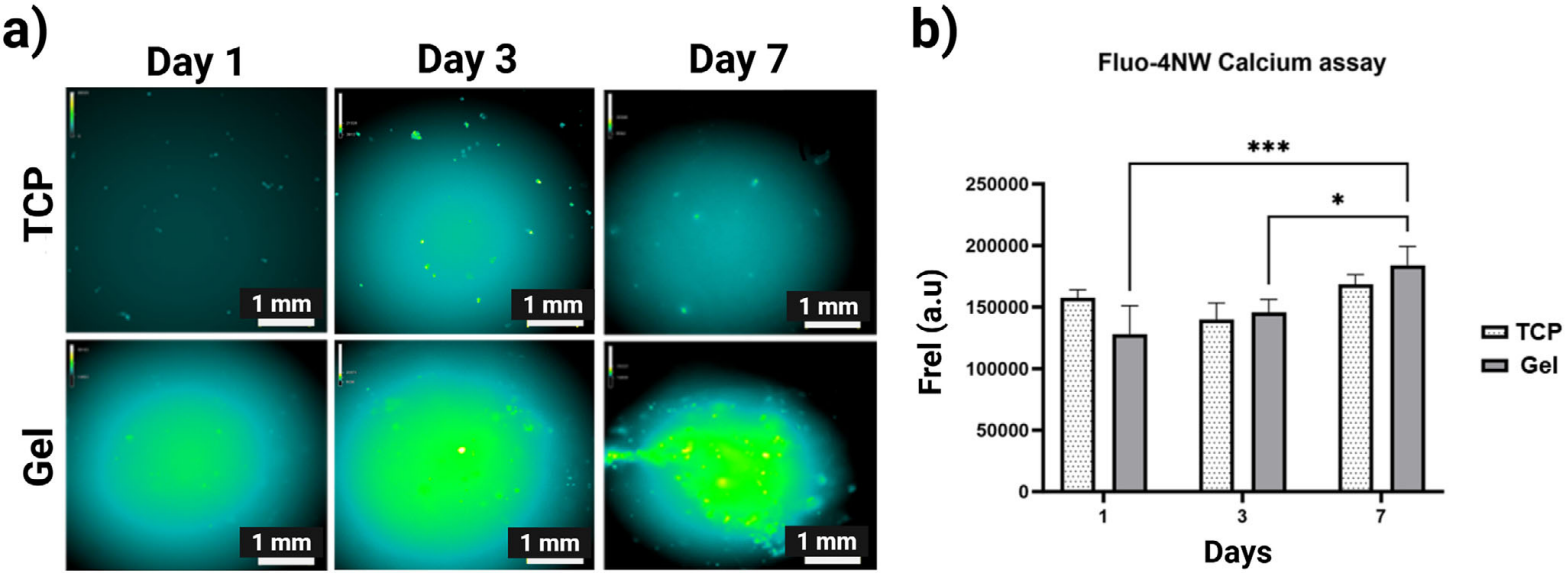
Calcium flux analysis, representative of changes in intracellular calcium concentration in HL‐1 on TCP (2D) and bioprinted within CAF (Gel). (a) Snapshot from time‐lapse video revealing the changes in fluorescent intensity between each image. The higher intensity was detected for Gel samples at all time points. Scale bar = 1 mm. (b) Calculation of the relative fluorescent intensity, indicating a significant increase between days 1 and 7 in the Gel, whilst TCP values are maintained; *n* = 3; (*) *p*<0.05; (***) *p*<0.001.

Functionality of HL‐1 cells was studied in terms of contractility, associated with fluctuations in calcium concentrations derived from their uptake and release by the cells. A slight increase in the fluorescence for TCP samples was observed across the culture period, however with less intensity than that seen for the Gel samples which were progressively brighter up to day 7 (Figure 4). This is expected as Gel samples have more cells than the TCP. Across the study period, no significant difference between the relative fluorescent intensity of the 2D and 3D cultures was observed (Figure 4b), indicating that the calcium flux assay was equally sensitive in both cases, and so the extent of fluorescence is thus a meaningful comparison of calcium mobilization.

The videos obtained from this assay can be accessed on the Supporting Information (Videos S1–S6), which display the intracellular calcium mobilization in real‐time, for both Gel and TCP, after 1, 3, and 7 days in culture.

#### 3.1.4 Confirmation of Cardiac Cell Functionality and Electrophysiology Assessed by MEA

**FIGURE 5:**
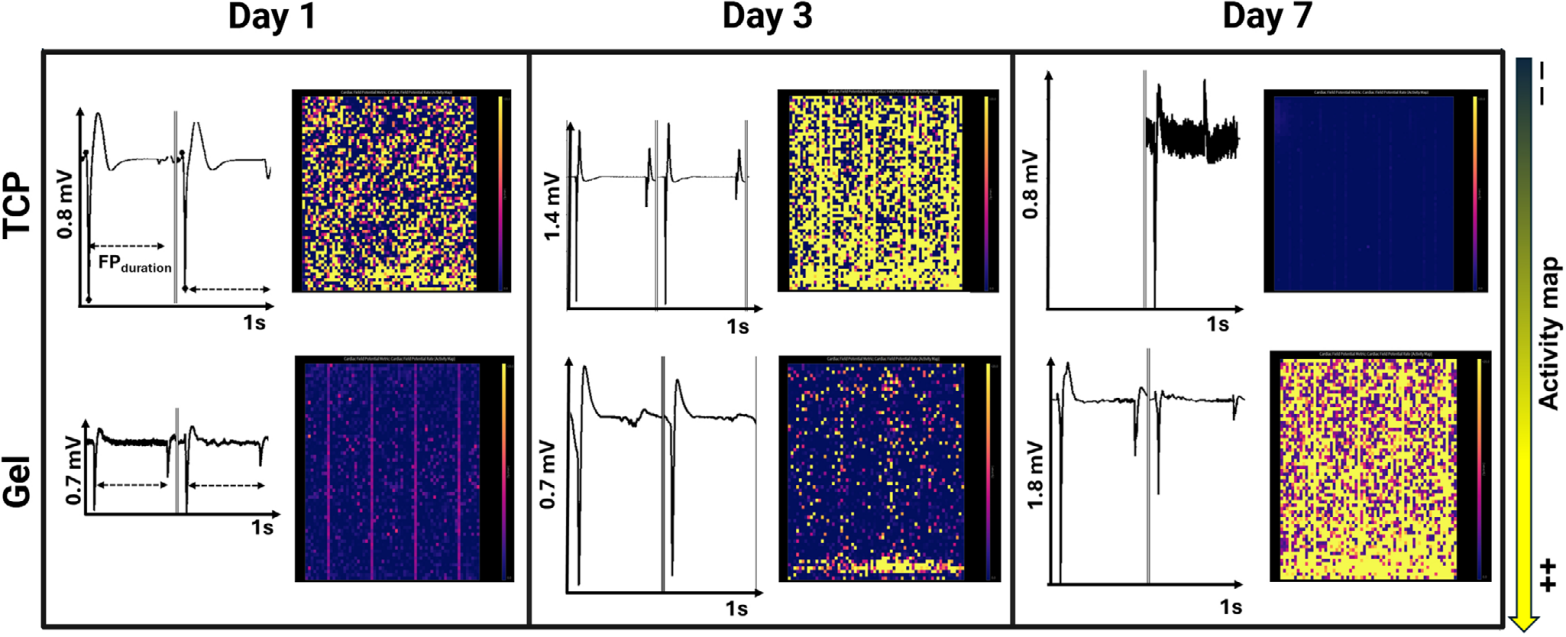
Representative field potential curves from MEA measurement of HL‐1 cells on hydrogel compared to TCP control, supported by the respective activity map (dark blue‐low activity; yellow‐high activity).

Action potential measurements within the cultures (Figure 5) show that at day 1, both Gel and TCP samples generate a defined action potential, with a similar field potential, approximately 0.7 and 0.8 mV, respectively. The field potential increased at day 3 for TCP samples, reaching 1.4 mV, results that are in accordance with previous studies [18]. For Gel samples, the field potential remained 0.8 mV, but the output signal was more defined and consistent. By day 7, the field potential for cells seeded on TCP decreased to 0.8 mV, but was no longer generated consistently, showing an irregular beating pattern. For the Gel samples, the opposite was observed, where the field potential more than doubled, going from 0.8 to 1.8 mV, with a well‐defined repetitive wave pattern, suggesting consistent beating. These results were supported by the activity maps, where a strong activity (yellow) is reported on day 1 for TCP, which is then decreased by day 7 (mainly blue), while for the Gel, an opposite trend is observed.

### 3.2 Characterization of Cardiac Cell Function and Electrophysiological Profile in Long‐Term Cultures

**FIGURE 6:**
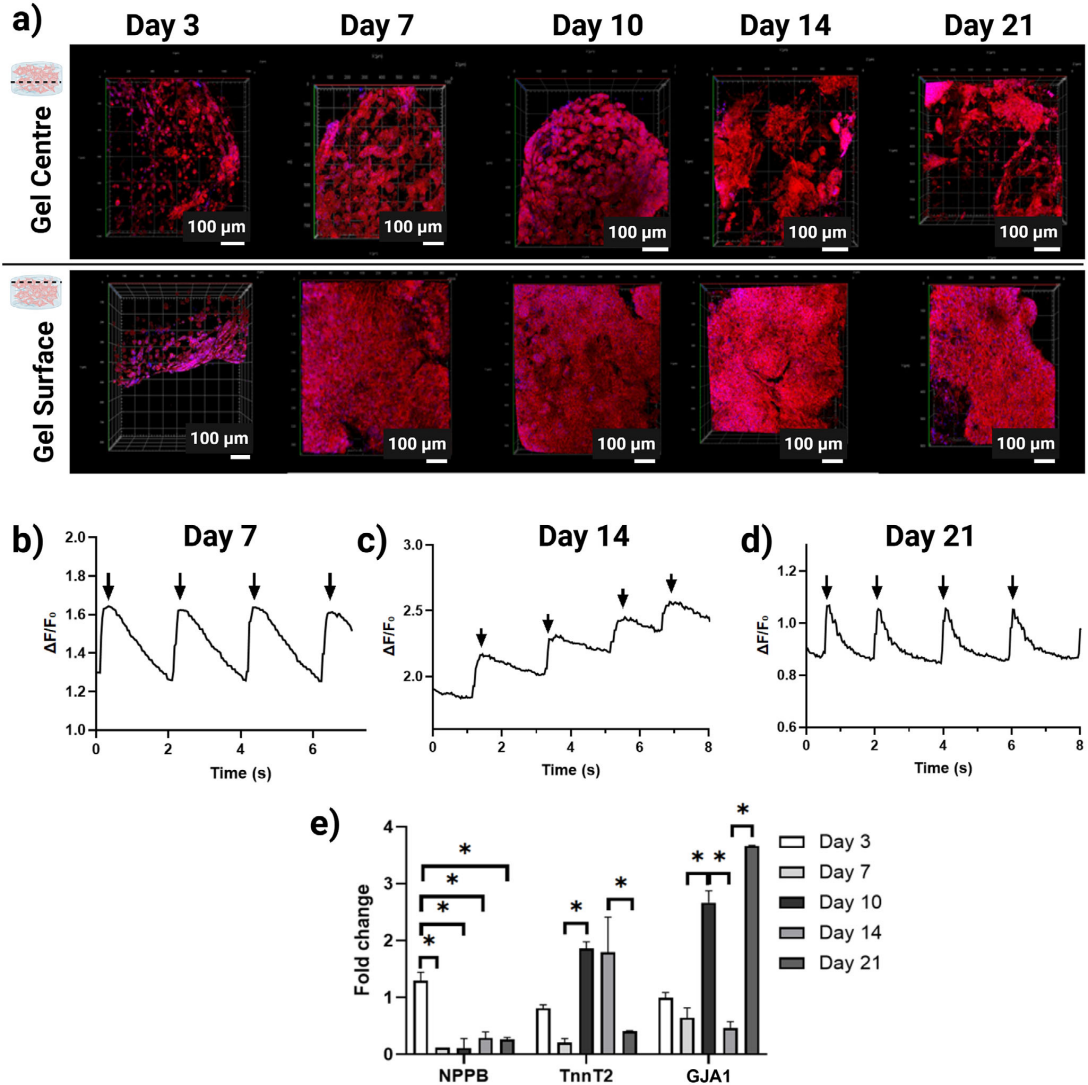
Extended culture of HL‐1 cells in CAF gel. (a) Immunostaining of HL‐1 cells in CAF gel with red depicting the actin filaments (Actin Red) and blue the cell nucleus (DAPI). Scale bar = 100 µm. (b–d) Representative plots of fluorescence fluctuations from the calcium flux assay on bioprinted HL‐1 cells at day 7 (b), 14 (c), and 21 (d). Arrows indicate the beating. (e) Confirmation of the genetic expression of relevant cardiac markers for HL‐1 cells: NPPB, TnnT2, GJA1; *n*=3; (*) *p* <0.05.

To further evaluate the long‐term behavior of the HL‐1 laden 3D bioprinted cultures, calcium flux and contractility were assessed after 7, 14, and 21 days of culture. Cell viability was confirmed after 3 days as per the previous experiment (Figure 2). The majority of cardiomyocytes were observed to be well‐populated and interconnected on the gel surface from day 7 through day 21 (Figure 6a). In the center of the gel cells cover most of the gel volume. Cell migration and hydrogel degradation promote a degree of clustering of cells in the center of the gel, which supports nutrient transport and oxygen diffusion. Intracellular calcium activity was monitored at days 7, 14, and 21 and visualized as temporal fluorescence changes (Figure 6b–d). These recordings demonstrate the persistence of spontaneous calcium transients over the 21‐day culture period, indicating maintained electrophysiological activity. Consistent calcium oscillations were observed across all time points, supporting sustained cellular excitability; however, fluorescent intensity was used solely as a qualitative indicator of calcium flux and was not interpreted as a direct measure of contractile strength.

The expression of key functional genes for cardiac tissue over time was seen from the expression of NPPB, TnnT2, and GJA1, using day 1 samples as a comparison. For NPPB, a slight upregulation was seen at day 3, followed by a downregulation up to day 21. The expression of TnnT2 did not change during the first week of culture (up to day 7); however, a significant upregulation was registered in the second week (days 10 and 14), with both time points reporting a fold change of approximately 2. This upregulation was not sustained, decreasing to baseline values by day 21. The trend observed for GJA1 was less constant, adopting a wave pattern from day 10, when this gene became upregulated (∼2.5 fold). This was followed by downregulation at day 14 (∼0.4), and a subsequent upregulation at day 21 (∼3.6).

### 3.3 Confirmation of Response of Bioprinted HL‐1 Cardiomyocytes to Drug‐Induced Dysrhythmia

**FIGURE 7:**
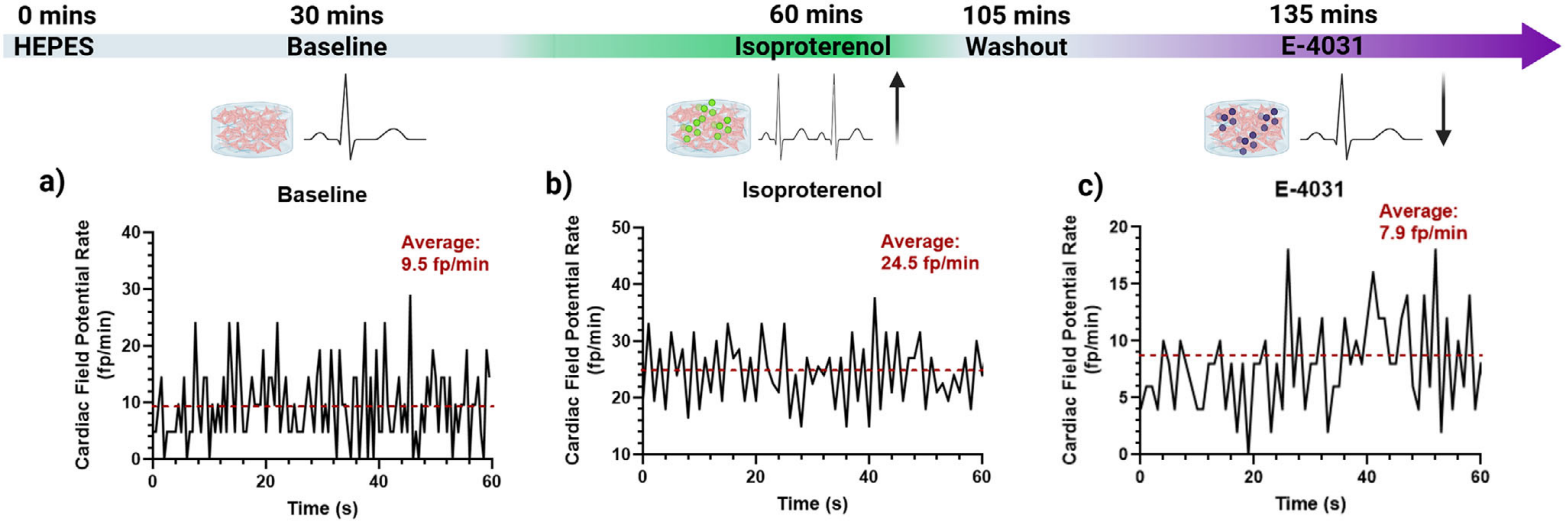
Drug response of bioprinted HL‐1s cultured for 7 days. Measurements were performed on untreated samples, followed by the addition of the β‐adrenergic agonist Isoproterenol at 30 min, and the hERG channel blocker E‐4031 at 135 min. (a–c) Representative plots of cardiac field potential rate of bioprinted HL‐1 cells measured from MEA untreated, and then with exposure to Isoproterenol and E‐4031.

For constructs exposed to β‐adrenergic receptor agonist isoproterenol and the cardiac hERG potassium channel blocker E‐4031, the field potential rate (fp/min) was measured at baseline, then with Isoproterenol, then with E‐4031 (Figure 7a–c). At baseline (Figure 7a), HL‐1 cells exhibited spontaneous electrical activity, with an average field potential rate of 9.5 fp/min, reflective of their intrinsic pacemaking capability, which served as a foundational reference for evaluating subsequent drug‐induced modulation. Upon administration of 100 nM of Isoproterenol, a significant increase in the average field potential rate was observed, 24.5 fp/min (Figure 7b), consistent with its β‐adrenergic agonist activity that augments cardiac beating rate and calcium cycling. Following washout of Isoproterenol, the cardiac field potential decreased to ∼ 8.5 fp/min, confirming the efficient removal of Isoproterenol (Figure S4). Subsequently, exposure to 50 nM of E‐4031 resulted in a reduction in field potential rate relative to baseline conditions, averaged at 7.9 fp/min (Figure 7c). This decrease suggests a prolongation of repolarization and disrupted beating rate of HL‐1 cells, characteristic of IKr inhibition.

## 4 Discussion

Drug discovery and development is a costly and challenging process, often inefficient, with each new compound taking up to 15 years to reach the market [25]. This is directly related to high failure and attrition rates during preclinical and clinical development, with drug‐induced cardiotoxicity being a major reason. Consequently, the development of predictive in vitro cardiac models remains a critical step toward improving the preclinical assessment of cardiotoxicity and drug efficacy. In this context, our study demonstrates that ReJI bioprinting—a high‐resolution and rapid deposition technique—can generate functional 3D cardiac constructs from HL‐1 cardiomyocytes, exhibiting a cardiac phenotype, with maturity of the constructs indicated by sustained spontaneous contractility for prolonged periods of up to 21 days.

### 4.1 Suitability of the CAF Hydrogel and ReJI Bioprinting Process for Fabrication of HL‐1‐Laden 3D Constructs

In bioprinting, the selection of an appropriate bioink is a critical determinant of both printing fidelity and subsequent cellular performance [26]. In cardiac tissue engineering (CTE), the ECM not only maintains construct architecture and guides cellular organization but also contributes to contractility and electrophysiological coupling. Building on this principle, we established a highly functional 3D cardiac construct by ReJI bioprinting HL‐1 cardiomyocytes for the first time within a well‐characterized hydrogel, made of type I collagen, alginate, and fibrin (CAF) [16]. Each of these natural polymers is widely used in CTE due to their biocompatibility and ECM‐mimicking properties; however, their combination as a tri‐component matrix has not been previously reported for supporting cardiomyocyte growth and function. Collagen provides cell‐adhesion motifs and tensile strength, alginate contributes to printability and structural integrity, while fibrin promotes fast crosslinking, cellular anchorage, and remodeling, ultimately generating a mechanically and biologically favorable environment for contractile tissue formation [27, 28]. Indeed, studies by Kaiser et al. investigated type I collagen‐fibrin hydrogel formulations for CTE, assessing the individual contributions of each protein toward scaffold stiffness and how these change cell behavior [28, 29]. In this study, we use the contractile HL‐1 cardiac cell line, derived from mouse atrial cardiomyocytes. This cell line typically requires culture on a gelatin/fibronectin‐coated substrate to ensure adequate attachment and cellular function. In our 2D control samples (TCP), this coating supported robust cell adhesion, viability and survival (Figure 2). In contrast, wells containing bioprinted HL‐1 were intentionally left uncoated to evaluate the intrinsic adhesive properties of CAF. After 7 days of culture, a continuous layer of interconnected HL‐1 cells was observed in these wells, even in the absence of coating (Figure S5). The integration and sustained adhesion of cells at the gel–substrate interface demonstrate that CAF possesses inherent bioactivity suitable for cell attachment, functioning effectively as a natural coating matrix and thus representing a viable alternative to gelatin/fibronectin formulations. Within CAF, HL‐1 cells remained viable at all time points for both short‐term and long‐term cultures, confirming the cytocompatibility of the hydrogel and its suitability for both embedded and surface cultures. The compression modulus of the CAF hydrogel is in the range of 10−20 kPa [30], which is at the lower end of the modulus range reported for healthy human cardiac tissue (<50 kPa [31]), but is still physiologically relevant. Future work will consider approaches to material formulation which raise the stiffness to match fibrotic tissue.

In bioprinting, extrusion‐based technologies (EBT) dominate the CTE field, but EBT processes can struggle with substrates which are rough or uneven when depositing gels which are structurally stable (rather than low viscosity). “Dragging” of the extruded material on the substrate can lead to fractures in the deposited tracks and uneven bioink deposition. High viability and retention of function have been reported across a range of cell types, seeding densities, and substrates with ReJI [12, 14, 30, 32]. As demonstrated herein, HL‐1 cells were successfully bioprinted with ReJI, demonstrating that the combination of CAF and ReJI offers a promising approach for the fabrication of viable 3D cardiac constructs.

### 4.2 Effects of 3D Culture on HL‐1 Morphology and Biological Function

The organization and functional performance of engineered cardiac tissues are greatly influenced by the cellular microenvironment and spatial configuration. In developing the 2D and 3D seeding strategies in this study we sought to maintain a similar average cell–cell center distance in tests. Seeding 125,000 cells in 12‐well plate and 5 million cells/mL in a 3D volume both equate to an average cell–cell center distance in the range of 55–60 µm. The 2D/3D comparisons are based on differences in spatial configuration and on variations in the proteins in culture (2D culture with gelatin and fibronectin‐coated substrates, and 3D culture with collagen, alginate and fibrin in the 3D hydrogel), but not differences in cell density. Cell–cell and cell‐ECM interactions are key for tissue function, establishment of synchronous contractility, appropriate cytoskeletal alignment, and representative cellular physiology. Reproducing these conditions in vitro represents a critical step toward developing predictive cardiac models for drug testing and disease modelling [33]. HL‐1 cells are derived from murine atrial cardiomyocytes and reportedly exhibit a mononucleated cardiac cell morphology in culture [17, 34, 35, 36]. Immunofluorescence staining and SEM of the cell cytoskeleton and nucleus (Figure 3) confirmed this cell morphology was not altered in the initial development of the hydrogel cultures. The production of sarcomeric myosin heavy chain and the intermediate filament protein desmin are fundamental to establishing a cardiomyocyte phenotype [37]. Similarly, myosin heavy chains are the motor proteins of the thick filaments in sarcomeres, generating contractile force, essential for cardiomyocyte contractility, mechanical coupling, and energetic homeostasis in cardiac cells [38, 39, 40, 41]. The sustained expression of myosin and desmin in HL‐1 cells in the 3D model in this study confirmed retention of the structural machinery required for synchronous contraction and the supportive cytoskeletal framework that underpins long‐term mechanical stability and maturation. For both TCP and Gel samples, there was a clear increase in the presence of both myosin and desmin throughout the 7 days of culture, suggesting an enhanced maturation of the cardiomyocyte phenotype, improved electromechanical coupling and greater resilience to the mechanical stresses inherent in engineered tissues [31, 42, 43, 44, 45].

The coordinated expression of myosin and desmin aligns with GJA1 upregulation (Figure 3c) observed in Gel samples. GJA1 is a key gap‐junction protein in the heart, and its expression and localization are closely linked to functional electromechanical coupling in cardiomyocytes [46]. An upregulation of this gene at day 1 indicates the onset of robust cell–cell communication via gap junction formation, essential for the propagation of electrical impulses and synchronized contractile behavior across the tissue [47, 48]. However, once coupling is established, a transient peak in GJA1 expression may give way to a more regulated, steady‐state level of connexin‐43, which is also observed for the Gel samples, whereby on day 7, GJA1 expression is downregulated compared to TCP. Interestingly, this pattern coincided with a decrease in NPPA expression at day 1 (Figure 3c), typically abundant in the atrial and ventricular myocardium during early cardiac development, decreasing as the tissue matures and remaining low but present in the atrium [49]. In addition, NPPA is upregulated under pathological stress, thus supporting the suitability of CAF to culture HL‐1 cells [49].

To confirm whether GJA1 stabilizes and whether functional phenotype is maintained, cultures were extended to 21 days (Figure 6c). A temporal regulation of GJA1 expression in Gel samples, observed over the 21‐day culture period, reflected a dynamic remodeling of intercellular communication and progressive tissue maturation. Compared to day 1 gel samples, a downregulation at days 3 and 7 suggests a phase of structural reorganization in which cells establish initial contact points and adapt to the 3D microenvironment. This early decrease has been similarly reported in other engineered cardiac tissues, where GJA1 expression transiently drops during the transition from monolayer to 3D culture, likely due to changes in cytoskeletal tension and ECM remodeling [50, 51]. By day 10, GJA1 was upregulated (∼3‐fold), indicating the reinforcement of gap junction networks and functional electrical coupling as the construct matured. Fluctuations in GJA1 expression thereafter, downregulation at day 14 and re‐upregulation by day 21, may correspond to periodic cycles of matrix remodeling and contractile adaptation, as described in other long‐term cultures of cardiomyocytes and cardiac organoids [52, 53]. The expression of NPPB was also assessed, as it is typically upregulated in cardiomyocytes under pathological stress, hypertrophy, or hypoxia [54, 55]. In our study, NPPB remained consistently downregulated throughout the 21 days of culture, indicating that the constructs did not activate a stress‐related response, already inferred from the downregulation of NPPA (Figure 3c).

In addition to the dynamic regulation of GJA1 and NPPB, the expression profile of TnnT2, a key marker of sarcomeric assembly and contractile maturity, provided further insight into the temporal evolution of HL‐1 phenotype within the 3D printed constructs. TnnT2 expression remained stable during the first week of culture, consistent with an early stage of structural organization and the initial establishment of cell–cell connectivity mediated by GJA1. However, by day 10 and day 14, a significant upregulation (∼twofold) in TnnT2 was observed, indicating enhanced sarcomeric organization and contractile apparatus development as cells transitioned toward a more mature functional state [53, 54, 56]. This period corresponded to the phase where intracellular calcium flux amplitudes increased, and electrical activity became more coordinated across the construct (Figure 6c,d), suggesting a tight coupling between transcriptional activation of the contractile apparatus and electrophysiological synchronization. By day 21, TnnT2 expression returned to baseline levels, while GJA1 expression increased. Together with the observed decline in electrical activity in calcium flux recordings, these results likely reflect a remodeling phase or adaptive response of HL‐1 cells within the 3D matrix, potentially associated with nutrient diffusion limitations or local hypoxia, both known to modulate sarcomeric gene expression and electrical conductance in vitro. Similar transient upregulation patterns of TnnT2 have been reported during cardiac tissue maturation in engineered constructs, where peak expression corresponds to optimal contractile performance, followed by stabilization or reduction under prolonged static culture [57, 58].

### 4.3 Electrophysiological Activity in a 3D Environment Over Time

A key hallmark of functional cardiac tissue is the ability to generate and propagate coordinated contractile activity driven by tightly regulated excitation‐contraction coupling. This process depends on both cell‐intrinsic properties (such as ion channel expression, calcium handling, and cytoskeletal organization) and extrinsic cues from the surrounding ECM. In this study, we examined the contractile and electrophysiological performance of HL‐1 cardiomyocytes cultured in both 2D and 3D environments to evaluate the impact of dimensionality, ECM composition, and cell density on functional maturation. Calcium mobilization was used as a primary indicator of excitation‐contraction coupling, while MEA analysis provided a quantitative assessment of electrical activity, including action potential amplitude, frequency, and propagation dynamics. In short‐term culture, TCP samples appeared to have an increase in fluorescence, indicative of increased calcium flux, peaking at day 3 (Figure 4a), followed by a decrease; however, measurements of the relative fluorescence intensity (Figure 4b), indicated no significant differences between timepoints. For Gel samples, a progressive increase in calcium flux fluorescence was observed in the recorded videos, reflected in relative fluorescence measurements. This likely reflects the combined effect of improved cell–cell interaction, which enhances intercellular coupling and calcium handling efficiency.

For extended culture periods (Figure 6b‐d), calcium flux traces initially displayed minimal regional variability, confirming spatially homogeneous beating and consistent contractile behavior throughout the construct. Both beating frequency and inter‐beat interval were stable, and intracellular calcium flux amplitudes were uniform, suggesting efficient intercellular coupling and calcium reuptake at this stage [59, 60]. By day 14, calcium flux profiles began to diverge, with noticeable alterations in shape and amplitude. Although overall spatial uniformity was maintained, the flux amplitudes became more heterogeneous, and recovery to baseline was incomplete following each beat. This partial recovery may indicate early signs of altered calcium cycling, possibly related to changes in sarcoplasmic reticulum calcium handling or shifts in GJA1 expression, previously observed at this stage. By day 21, constructs displayed a distinct intracellular calcium flux profile, with larger amplitude oscillations and the emergence of post‐beat “undershoots,” where intensity transiently fell below pre‐beat baseline levels, typically associated with hyperactive or asynchronous calcium reuptake. Spatial uniformity decreased, reflected by greater regional variability in flux intensity, suggesting the onset of localized functional heterogeneity within the construct. Although beating frequency increased relative to earlier time points, beat intervals and intracellular calcium flux amplitudes were more variable, indicating a dynamic but less synchronized contractile behavior [61].

The observed temporal changes in intracellular calcium flux profiles align closely with the dynamic regulation of GJA1 expression throughout the culture period, suggesting an active remodeling of gap junctions as the constructs mature. Briefly, at early stages, reduced GJA1 levels may limit electrical coupling, leading to the initial establishment of local synchronization within clusters of cells, while a later upregulation facilitates the restoration of global connectivity and more efficient calcium wave propagation across the tissue [62]. The partial desynchronization and spatial heterogeneity observed by day 21 may therefore reflect structural and electrophysiological remodeling processes that naturally occur in long‐term cardiac cultures, as gap junctions undergo turnover and redistribution in response to mechanical loading and metabolic changes [63]. These results indicate that the HL‐1 constructs not only sustain functional contractility over extended culture but also undergo adaptive electrical maturation, where GJA1‐mediated coupling and calcium handling mechanisms evolve to support long‐term physiological activity in a 3D environment.

Complementary to calcium imaging, MEA analysis was used to monitor the electrophysiological activity of HL‐1 cells cultured in 2D and 3D environments, providing insight into action potential dynamics and network synchronization over time (Figure 5). Previous work by De Santis et al. demonstrated that increasing initial cardiomyocyte density significantly accelerates the onset of contractile activity, even if initially uncoordinated, with functional organization improving as proliferation and cell alignment progress [64]. In the present study, spontaneous contractility was observed as early as 24 h post‐printing, underscoring the benefits of high‐density seeding and the supportive microenvironment of the CAF hydrogel in facilitating rapid establishment of electrically coupled cardiac‐like tissue. In TCP cultures, cells exhibited a stable and well‐defined action potential from day 1; however, by day 7, both the frequency and definition of electrical spikes were markedly reduced, indicating a loss of coordinated electrical activity and the onset of culture deterioration, a phenomenon previously reported for HL‐1 cells in 2D culture, for 5–8 days [18]. In contrast, Gel samples demonstrated sustained electrical activity across the full culture period with a doubling in the membrane potential between day 1 and 7, accompanied by homogeneous activity maps displaying widespread, synchronized electrical activation across the sample. This enhancement strongly supports the role of high cell–cell contact and the 3D environment in promoting effective coupling and functional maturation.

For cultures maintained beyond seven days, MEA recordings revealed a gradual decline in both the amplitude and spatial uniformity of electrical activity, accompanied by reduced signal intensity in the activity maps (Figure S6). The decrease in MEA signal amplitude after one week likely represents a transition from early, highly synchronized electrical activity toward a more heterogeneous and compartmentalized conduction network, characteristic of developing cardiac tissue rather than loss of viability [17, 63, 65]. Moreover, the persistent downregulation of NPPB across all time points suggests that these changes are not indicative of a stress response but further confirm the intrinsic electrophysiological remodeling within the 3D constructs as they mature.

Whilst murine, HL‐1 cells were selected for this study as they are the only immortalized cardiomyocyte cell line available. HL‐1 cells are long established, well validated and have previously been used to study cardiac signaling, electrical coupling, and transcriptional regulation [34] and bioelectronic monitoring of cardiac electrophysiology [66]. Whilst they do not precisely replicate human cardiomyocyte behavior they are qualitatively very similar in their electrophysiological response. The human alternatives to HL‐1 cells would be either primary cardiomyocytes or induced pluripotent stem cell (iPSC) derived cardiomyocytes. The number of cells that our 3D models require would be challenging for use of primary cells. Use of iPSC‐derived cardiomyocytes has potential, but with extended culture times, and will form part of future work.

### 4.4 Drug‐Induced Modulation of Contractile and Electrical Activity in 3D Bioprinted HL‐1 Cardiac Constructs

To assess the physiological responsiveness and pharmacological relevance of the bioprinted HL‐1 constructs, two model compounds with known effects on cardiac electrophysiology were employed: Isoproterenol and E‐4031. These two agents are widely used in cardiotoxicity screening to evaluate the functional maturity and predictive accuracy of cardiac in vitro models. Here, the differential drug responses obtained further validated the functional maturity and physiological relevance of the ReJI‐bioprinted HL‐1 constructs (Figure 7). Exposure to Isoproterenol elicited a pronounced positive chronotropic response, evident from the increased field potential rate. This outcome is consistent with its known β‐adrenergic agonist activity, which enhances cardiac contractility through increased calcium influx, thereby augmenting both beating frequency and contraction amplitude [67, 68]. Following E‐4031 administration, a decline in field potential rate is observed, characteristic of hERG channel inhibition, which prolongs repolarization [69, 70, 71]. The reduction in spontaneous beating likely reflects delayed action potential termination and impaired calcium expulsion, consistent with potassium channel blockade. Importantly, these effects with both drugs occurred without evidence of construct degradation or cytotoxicity, indicating that the HL‐1 tissues maintain structural integrity under electrophysiological perturbation.

## 5 Conclusions

This study demonstrates that ReJI‐based 3D bioprinting enables the fabrication of highly viable and functional HL‐1 cardiac constructs capable of maintaining physiological phenotype, protein expression, and contractile behavior over extended culture periods. The constructs exhibited robust cellular organization, enhanced cell–cell and cell–ECM interactions, and sustained expression of key cardiac markers, including myosin and desmin. Electrophysiological analyses further confirmed the development of a mature and responsive cardiac phenotype, with an organized electrical activity and progressive contractile maturation, ultimately aligning with the natural remodeling behavior of HL‐1 cells in vitro. Importantly, exposure to drugs modulating cardiac cell electrophysiology elicited predictable dysrhythmic responses, qualifying the model's ability to reproduce clinically relevant electrophysiological outcomes and detect drug‐induced toxicities. Collectively, these findings underscore the advantages of 3D bioprinted cardiac constructs over conventional 2D models, particularly in their capacity to replicate native‐like cellular architecture, intercellular coupling, and electrophysiological behavior in a controlled and reproducible platform. The integration of high‐throughput compatible readouts, such as calcium imaging and MEA recording, further strengthens the translational potential of this system. Future work will focus on extending this approach to multicellular cardiac models incorporating cardiac fibroblasts and endothelial cells, integrating mechanical stimulation to achieve long‐term maturation and physiologically accurate contractility, and considering the potential for integration of human cardiomyocytes derived from induced pluripotent stem cells.

## Author Contributions

**Kenneth Dalgarno** conceptualized the study, obtained funding, resources, and administered the project. All authors contributed to methodology. **Priscila Melo**, **Wing Tai Tung**, **Harvey Lewis**, **Kavin Hettiarachchilage**, **Babis Tzivelekis**, and **Rolando Berlinguer‐Palmini** undertook the investigation. Kenneth Dalgarno, Priscila Melo, Wing Tai Tung, **Ana Marina Ferreira**, and **Jason Gill** supervised elements of the research. All authors contributed to drafting the manuscript. Kenneth Dalgarno, Priscila Melo, Wing Tai Tung, Rolando Berlinguer‐Palmini, Ana Marina Ferreira and Jason Gill reviewed and edited the manuscript .

## Funding

This project has received funding from the (i) UK EPSRC through award EP/V013092/1, (ii) European Union's Horizon Europe research and innovation program under grant agreement No. 101091852, and (iii) National Centre for the Replacement, Refinement and Reduction of Animals in Research (NC3Rs) through award APP46911.

## Conflicts of Interest

The reactive jet impingement bioprinting process used within this paper has been patented, and Dalgarno has patent #WO2019008373 issued to Newcastle University. The patent has been conditionally assigned to a spin‐out company called Jetbio. Dalgarno, Melo, Ferreira‐Duarte, and Tzivelekis are all shareholders in Jetbio.

## Supporting information




**Supporting File 1**: adhm71446‐sup‐0001‐SuppMat.docx.

**Supporting File 2**: adhm71446‐sup‐0002‐FigureS1‐S6.zip.

**Supporting File 3**: adhm71446‐sup‐0003‐VideoS1‐S6.zip.

## Data Availability

Data supporting the findings of this study are openly available in the Newcastle University Research Repository at doi: https://doi.org/10.25405/data.ncl.30752198.
