# Supplementary material for: Bioprinting High‐Cell‐Density Cardiac Tissue Constructs With Sustained Contractile Function
Source: Adv Healthc Mater. 2026 Jul 19;15(31):e71446. doi: 10.1002/adhm.71446 (PMC13495887; doi:10.1002/adhm.71446)
Supplement: Supplementary file 1 [file ADHM-15-0-s003.docx]

**Supplementary Material**

**Figure S1 –** Analysis Workflow for MEA Test Data

**Figure S2 –** Separated Green and Red Fluorescent Protein Channels from Live/Dead Analysis in Figure 2(b).

|  |  |
| --- | --- |
| **Figure S3** – Separated Fluorescent Channels for Figure 3(a).  Imaging of Desmin on Left, and Myosin on Right. | |

**Figure S4 –** Drug response of bioprinted HL-1 cultured for 7 days. MEA measurements were performed on untreated samples, followed by the addition of the β-adrenergic agonist Isoproterenol at 30 minutes, then a washout period of 45 minutes, and finally the addition of the hERG channel blocker E-4031.

**Figure S5 –** Immunostaining of HL-1 cells after 7 days of culture, growing in the uncoated TCP. Red depicts the actin filaments (Actin Red) and blue the cell nucleus (DAPI). Scale bar is 300 µm.

**Figure S6 –** Representative field potential curves from MEA measurement of HL-1 cells on hydrogel compared to TCP control, supported by the respective activity map (dark blue - low activity; yellow - high activity). Left Image: Day 7; Right Image: Day 10.
